# SuhB Regulates the Motile-Sessile Switch in *Pseudomonas aeruginosa* through the Gac/Rsm Pathway and c-di-GMP Signaling

**DOI:** 10.3389/fmicb.2017.01045

**Published:** 2017-06-08

**Authors:** Kewei Li, Guangjian Yang, Alexander B. Debru, Pingping Li, Li Zong, Peizhen Li, Teng Xu, Weihui Wu, Shouguang Jin, Qiyu Bao

**Affiliations:** ^1^Institute of Biomedical Informatics, School of Laboratory Medicine and Life Science, Wenzhou Medical UniversityWenzhou, China; ^2^State Key Laboratory of Medicinal Chemical Biology, Key Laboratory of Molecular Microbiology and Technology of the Ministry of Education, Department of Microbiology, College of Life Sciences, Nankai UniversityTianjin, China; ^3^Department of Molecular Genetics and Microbiology, College of Medicine, University of Florida, GainesvilleFL, United States

**Keywords:** SuhB, motility, biofilm, Gac/Rsm, c-di-GMP, lifestyle switch, *P. aeruginosa*

## Abstract

Many *Pseudomonas aeruginosa* virulence traits that contribute to human infections are accepted as being associated with its environmental lifestyle. Therefore, identifying the molecular mechanisms that govern the lifestyle choice is of high significance. We previously reported that a mutation in *suhB* results in a decrease in swimming motility and increased biofilm formation compared to the wild-type strain. Yet, little is known about how this occurs. In this study, we demonstrated that SuhB inversely regulates motility and biofilm formation through the GacA-RsmY/Z-RsmA cascade. Mutations in *gacA* or the two small RNAs *rsmY*/*rsmZ*, or overproduction of the RsmA protein essentially rescued the motility defect of the *suhB* mutant. Additionally, we identified a c-di-GMP mediated mechanism for SuhB regulation of motility and biofilm formation. We showed that the Δ*suhB* mutant displayed elevated levels of c-di-GMP, and the Δ*suhB* motility and biofilm phenotypes could be switched by artificially decreasing c-di-GMP levels. Further experiments led to the identification of the diguanylate cyclase GcbA responsible for regulating the c-di-GMP concentration in Δ*suhB* and hence the switch between planktonic and surface-associated growth. Together, our results demonstrate a novel mechanism for SuhB regulation of the lifestyle transition via the Gac/Rsm and c-di-GMP signaling networks in *P. aeruginosa*.

## Introduction

Many bacteria are able to adopt two different lifestyles. While a free-living state enables dissemination and exploration of novel niches, growing as a surface-attached community offers protection and survival in unfavorable environments (O’Toole, 2004; Flemming et al., 2016). The successful transition between motile and sessile lifestyles is crucial to their ecological success, and requires a sophisticated regulatory network to sense and integrate various environmental cues into an appropriate response (Kolter and Greenberg, 2006; Krell et al., 2010). Therefore, understanding the regulatory mechanisms that govern the lifestyle switch is of prime importance and may provide potential target for combating bacterial infections.

*Pseudomonas aeruginosa* is a major opportunistic human pathogen that causes numerous acute and chronic infections (Driscoll et al., 2007; De and Plésiat, 2011). Recent data indicate that the motile/toxic or sessile/biofilm lifestyle of *P. aeruginosa* directly relates to the acute or chronic infection mode (Coggan and Wolfgang, 2012). *P. aeruginosa* swimming motility is powered by the rotation of a single, polar flagellum (Rashid and Kornberg, 2000). In addition, flagella and/or motility could also contribute to the early attachment of biofilms (O’Toole and Kolter, 1998), which are a common cause of persistent and chronic infections (Bjarnsholt, 2013).

Two-component signal transduction systems (TCS) have been implicated as key mediators of *P. aeruginosa* lifestyle (Coggan and Wolfgang, 2012; Jimenez et al., 2012). A conserved and perhaps the most interesting TCS is referred to as the GacS/GacA system (Brencic et al., 2009). GacS is a transmembrane sensor kinase which phosphorylates GacA, and phosphorylated GacA exclusively activates the transcription of the two small regulatory RNAs RsmY and RsmZ (Brencic et al., 2009). A membrane-bound hybrid sensor, RetS, inhibits the GacS/GacA signaling by forming a RetS/GacS heterodimer, leading to the downregulation of RsmY/Z (Goodman et al., 2009). The function of these sRNAs is to sequester the RNA binding protein RsmA, a central post-transcriptional regulator which represses the production of sessile and biofilm determinants, while free RsmA leads to a planktonic and more virulent lifestyle (Costerton et al., 1999; Brencic et al., 2009).

In parallel to the GAC system, the second messenger cyclic-di-GMP (c-di-GMP) has recently emerged as a central regulator of the switch between lifestyles (Hengge, 2009; Romling et al., 2013). While high c-di-GMP levels correlate with a sessile lifestyle, low levels of this molecule are known to promote motility (Hengge, 2009). The levels of c-di-GMP are enzymatically controlled by diguanylate cyclases (DGCs) responsible for synthesis, and phosphodiesterases (PDEs) involved in degradation (Kulesekara et al., 2006). In *P. aeruginosa* genome, more than 40 genes encoding either a DGC or a PDE have been identified (Valentini and Filloux, 2016). This abundance suggests a network of pathways that feeds c-di-GMP into a common pool or, alternatively, an array of non-converging pathways that produce highly specific concentrations of c-di-GMP (Hengge, 2009).

Previously, we identified SuhB as a critical regulator of multiple virulence factors in *P. aeruginosa*, including the type III secretion system (T3SS), swimming motility, type VI secretion system (T6SS) and biofilm formation (Li et al., 2013). Transcriptome analysis revealed that SuhB-mediated regulatory pathways might partially overlap those under the control of RetS and RsmA (Goodman et al., 2004; Brencic and Lory, 2009; Li et al., 2013). Mutation of *suhB* leads to upregulation of GacA and its downstream small RNAs RsmY/Z, which then repress T3SS genes and trigger T6SS expression and biofilm formation (Li et al., 2013). It is worth mentioning that a connection between RetS and c-di-GMP pathways has also been suggested, whereas a *retS* mutant displays elevated levels of c-di-GMP in *P. aeruginosa* (Moscoso et al., 2011). On the other hand, we have recently demonstrated a role of SuhB in the regulation of resistance to aminoglycoside antibiotics by modulating ribosome activity (Shi et al., 2015). The relationships between SuhB and genes involved in distinctive pathways as well as the pleiotropic regulatory functions suggest SuhB play a role in global gene regulation.

Nevertheless, there are still many questions to be answered and asked about the SuhB regulon. It was shown that SuhB is involved in the inverse regulation of swimming motility and biofilm formation in *P. aeruginosa*, but the mechanism by which SuhB influences these processes remains unclear. Given the relationship between SuhB and Gac/Rsm system, and the partially overlapping regulons between SuhB and RetS, whose mutation exhibits increased levels of c-di-GMP, the present study aimed to investigate whether the Gac/Rsm and c-di-GMP signaling are required for the SuhB-mediated inverse regulation. By deleting or overexpressing specific components of the Gac/Rsm cascade, we demonstrate that this regulation is mediated partly by the Gac/Rsm pathway. Moreover, we identified a c-di-GMP mediated mechanism for SuhB regulation of biofilm formation and motility, and demonstrated that the DGC GcbA is a target of SuhB to modulate the c-di-GMP concentration responsible for regulating the transition from planktonic to surface-associated growth. Collectively, these results provide novel insight into the molecular mechanism underlying the SuhB-mediated regulation of motile-sessile transition.

## Materials and Methods

### Bacterial Strains, Plasmids, and Culture Conditions

The bacterial strains and plasmids used in this study are listed in Supplementary Table S1. *P. aeruginosa* strain PAK was used as the parental strain. *Escherichia coli* strain DH5α was used as a routine cloning host for plasmid construction. *P. aeruginosa* and *E. coli* strains were grown in Lysogeny broth (LB) medium (10 g of tryptone, 5 g of yeast extract, and 5 g of NaCl per liter, pH 7.0) with shaking or on LB Agar (LB medium containing 1.5% [w/v] agar) at 37°C unless otherwise noted. Antibiotics were used at the following concentrations where appropriate: 100 μg/ml gentamicin, 150 μg/ml carbenicillin and 50 μg/ml tetracycline for *P. aeruginosa* and 100 μg/ml ampicillin, 50 μg/ml kanamycin, 10 μg/ml gentamicin and 10 μg/ml tetracycline for *E. coli*.

### Construction of Strains and Plasmids

In-frame deletions of *suhB*, *gacA* and the double deletion *rsmY rsmZ* were previously constructed (Li et al., 2013), by allelic exchange employing the sucrose counter-selection system with the gene replacement vector pEX18Tc (Hoang et al., 1998). For engineering the *gcbA* (*PA4843*) deletion mutant, similar procedures were used except the different primers (Supplementary Table S2). Complementation and overexpression were accomplished by placing the respective genes under the control of the *lac* promoter in the pUCP20 vector. The plasmid pDN19*lacZ*W carrying a promoter-less *lacZ* reporter gene was used to construct promoter-*lacZ* reporter fusions of the *cdrA* and other genes as previously described (Sun et al., 2014).

### Swimming Motility Assay

Swimming motility was assessed on tryptone plates (10 g of tryptone, 5 g of NaCl per liter containing 0.3% [w/v] agar, pH 7.0) as previously described (Rashid and Kornberg, 2000; Amiel et al., 2010). Briefly, plates were inoculated with bacteria from overnight LB cultures by puncturing inoculates halfway through the depth of the agar with a sterile toothpick and incubated at 37°C for 18 h. The diameter of the circular turbid zone as an indication of swimming motility was measured in millimeters. Plates were subsequently photographed with a digital camera. The experiments were performed three times with three replicates each time.

Swimming motility was also monitored in 3% (w/v) Ficoll (low-viscosity mimicking swimming conditions) as described elsewhere (Toutain et al., 2005). Bacterial strains expressing ZsGreen1 green fluorescent protein (GFP) were visualized via fluorescence microscope (Eclipse Ni-U, Nikon) equipped with a 100×/1.45 oil objective. The GFP-expressing bacteria were generated by transformation of the indicated strains with a multicopy plasmid (pUCP-*zsGreen1*, GFP^+^) that constitutively expresses ZsGreen1 under the control of a P*_lac_* promoter (West et al., 1994). Approximately 100 cells were visualized for each strain.

### Transmission Electron Microscopy

Bacterial cultures were gently suspended in 2.5% (v/v) glutaraldehyde in 0.1 M PBS (pH 7.2) and the fixation process started at the same time. After 15 min, carbon-coated copper grids were dipped into a drop of the cell suspension for 30 s to allow bacterial adhesion. Excess liquid was eliminated with filter paper and grids were rinsed twice with distilled water and excess liquid was eliminated again. The grids were stained for 2 s with 2% (w/v) uranyl acetate, washed twice for 10 s in a drop of water and air-dried. The negatively stained cells were then visualized with a Hitachi H-7500 transmission electron microscope to confirm cell morphology and the presence of flagella.

### Determining Swim Reversals

Swim reversal rate measures the frequency at which a swimming cell changes its direction as previously described (Toutain et al., 2005; Caiazza et al., 2007; Petrova et al., 2014). Briefly, ZsGreen1-expressing *P. aeruginosa* strains were grown to exponential (OD_600_ = 0.3) and stationary phase (OD_600_ = 2.5), diluted 1:10–1:100 in 0.9% (w/v) NaCl containing 3% (w/v) Ficoll. Cells were observed via fluorescence on a Nikon Eclipse Ni-U microscopy with a 100×/1.45 oil objective, and real-time videos were captured using a Nikon DS-Fi1C camera, NIS-Elements F Ver4.00.00 and Camtasia Studio V7.5 software package. The videos were subsequently analyzed to monitor individual cells within the field of view for the number of changes in motility direction. Only cells remaining within the field of view for the duration of the video were considered. Approximately 50 cells were counted for each strain and reversal rates are reported as reversals per cell per minute.

### Measurement of c-di-GMP Levels

Quantification of c-di-GMP was adapted from procedures previously described (Rybtke et al., 2012; Moscoso et al., 2014;Lo et al., 2016), with the following modifications. Briefly, a plasmid-based c-di-GMP reporter was created by transcriptionally fusing the c-di-GMP-responsive *cdrA* promoter to the *lacZ* gene encoding the enzyme β-galactosidase. The *cdrA* (*PA4625*) promoter from *P. aeruginosa* PAK was amplified using the primer pair *cdrA*-LacZ-F and *cdrA*-LacZ-R (Supplementary Table S2). The amplified promoter was inserted between the *Eco*RI and *Bam*HI sites of the promoterless *lacZ* expression vector pDN19*lacZ*W. The intracellular levels of c-di-GMP were gauged by measuring the β-galactosidase activity, and data are presented as relative c-di-GMP levels which were normalized to the levels of the reference strains.

For β-galactosidase measurements, overnight cultures were diluted 1:100 into LB and the cultivation was continued for additional 3.5 h (Chen et al., 2016). The β-galactosidase activity was determined by the Miller method (Sambrook et al., 1989). All assays were carried out in triplicates and each assay was performed at least twice.

### Biofilm Analysis

Biofilm assay was performed as described (O’Toole and Kolter, 1998) with minor modifications. Briefly, overnight cultures were inoculated at a final OD_600_ of 0.0025 into LB medium in glass tubes or 24-wells polystyrene microtitre dishes and grown for 24 h. Initial attachment to a polystyrene surface was measured following 6 h of growth as previously described (Petrova et al., 2014). Then planktonic cells were removed and tubes were washed gently with water for three times. Biofilms were stained with 0.1% (w/v) crystal violet for 15 min and tubes were rinsed three times with distilled water. The tubes can be photographed when dry and for quantification of the biofilm crystal violet stain was solubilized in 95% (v/v) ethanol before measuring the absorbance at 600 nm.

For visualization of biofilm architecture, biofilms were grown on glass coupons in 24-well microtitre plates for 48 h under conditions described above. Then sample coupons were rinsed three times with PBS and stained with propidium iodide (60 μM) for 30 min at room temperature, protected from light. Excess stain was removed by rinsing the sample gently with sterilized water. Biofilm architecture was visualized via confocal laser scanning microscopy (CLSM) by using a Nikon A1 confocal microscope. The CLSM images acquisition and 3D reconstructions were done with the Nikon’s NIS-Elements AR software.

### Quantitative Real-Time Reverse-Transcription PCR (qRT-PCR)

Overnight bacterial cultures were subcultured in LB medium and grown to an OD_600_ of 1.0. Total RNA was extracted using the RNAprep pure Cell/Bacteria Kit according to the manufacturer’s protocol with on-column DNase I digestion (TIANGEN Biotech, Cat #DP430). RNA samples were further subjected to DNase I treatment and purified using the RNAclean Kit (TIANGEN Biotech, Cat #DP412). RNA concentration and purity were analyzed with an NanoDrop 2000 spectrophotometer (Thermo Scientific) and integrity was verified in denaturing agarose gels. cDNA was generated using the PrimeScript RT Reagent Kit (Perfect Real Time) (Takara, Cat #RR037A) according to the manufacturer’s instructions with random hexamers. Quantitative real-time reverse-transcription PCR (qRT-PCR) was performed using the SYBR^®^ Premix Ex Taq^TM^ (Tli RNaseH Plus) (Takara, Cat #RR420A) with the primers listed in Supplementary Table S2. The level of 30S rRNA *rpsL* was used as a control (Wu and Jin, 2005).

### Statistical Analysis

Statistical analysis was carried out using SPSS program and results were expressed as the mean values ± standard deviations. As indicated, one-way analysis of variance (ANOVA) followed by Tukey’s multiple comparison test or Student’s *t*-test analysis was performed to assess statistical significance of the data.

## Results

### Motility Defect of the *suhB* Mutant

**FIGURE 1:**
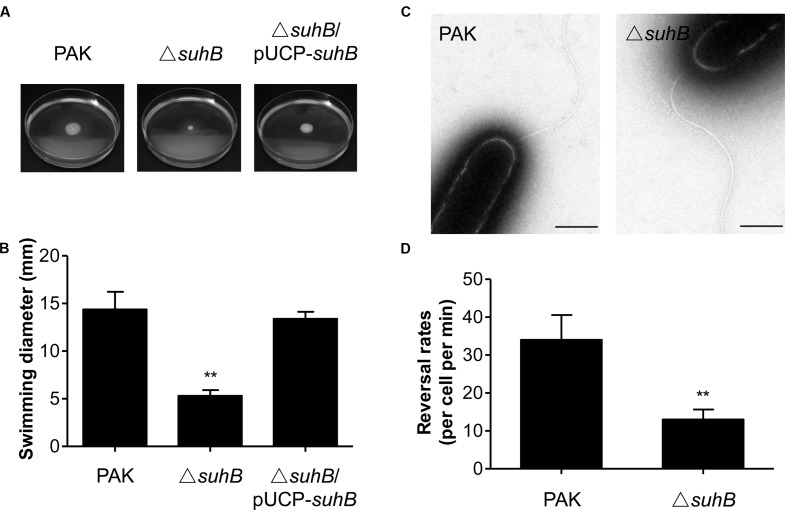
The *suhB* mutant is defective for swimming motility. **(A)** Cells were inoculated with a toothpick from an overnight Lysogeny broth (LB) culture onto tryptone soft agar plates and photographed after 18 h incubation at 37°C. **(B)** The diameters of swimming zones were measured from three experiments. ^∗∗^*P* < 0.01 compared to WT or complemented strains by Student’s *t*-test. **(C)** Transmission electron microscopy of wild-type PAK and the *suhB* mutant grown to stationary phase. Bar, 500 nm. **(D)** Flagellar reversal rates in 3% (w/v) Ficoll mimicking swimming conditions were measured as changes in movement direction of stationary-phase cells. Rates are expressed as reversals per cell per minute. Error bars denote standard deviations. ^∗∗^*P* < 0.01 compared to wild type PAK by Student’s *t*-test.

It has previously been shown that SuhB is required for both swimming motility and biofilm formation (Li et al., 2013), suggesting this protein might be involved in coregulating these processes. We hypothesized that *suhB* may serve as a genetic link between these phenomena and attempted to determine the molecular basis of this link in the current studies. We firstly pursued the flagella-mediated swimming motility using tryptone soft agar motility assay. In agreement with previous results on 0.3% LB agar (Li et al., 2013), the *suhB* mutant is defective in swimming on the tryptone soft agar plates, and complementation of the mutant with a *suhB*-expressing plasmid (pUCP-*suhB*) restored normal swimming (**Figures 1A,B**).

To characterize the swimming motility defect in more detail, cells constitutively expressing GFP were generated and observed by light microscopy in media with viscosity mimicking swimming (3% Ficoll) conditions (Toutain et al., 2005). In general, the mono-flagellated *P. aeruginosa* swims in a straight forward-backup/reversal-straight forward mode (Pratt and Kolter, 1998; Ping et al., 2013). The results showed that during exponential phase (OD_600_ = 0.3) about 35% of wild-type cells were motile and exhibited the run/back-up/run mode of swimming, whereas approximately 19% of the *suhB* mutant cells were also motile, but tended to swim straight and exhibited less frequent reorientations as compared with the wild-type. Interestingly, during stationary phase (OD_600_ = 2.5) the percentage of motile cells in the *suhB* mutant was comparable to that of the wild-type strain (75% for wild-type strain, and 70% for *suhB* mutant). Transmission electron microscopy (TEM) analysis of cells from stationary phase showed that the *suhB* mutant possessed apparently normal flagellum and the cell morphology was comparable to the wild-type PAK (**Figure 1C**). However, when the flagellar reversal rates (the frequency at which a motile cell changes its movement direction) was tested at stationary phase, inactivation of *suhB* resulted in a significantly reduced frequency of changes in cellular movement direction compared to those of the wild-type strain (**Figure 1D**), suggesting an abnormal function of flagella in the *suhB* mutant.

### SuhB Regulates Motility through the GacA-RsmY/Z-RsmA Regulatory Cascade

**FIGURE 2:**
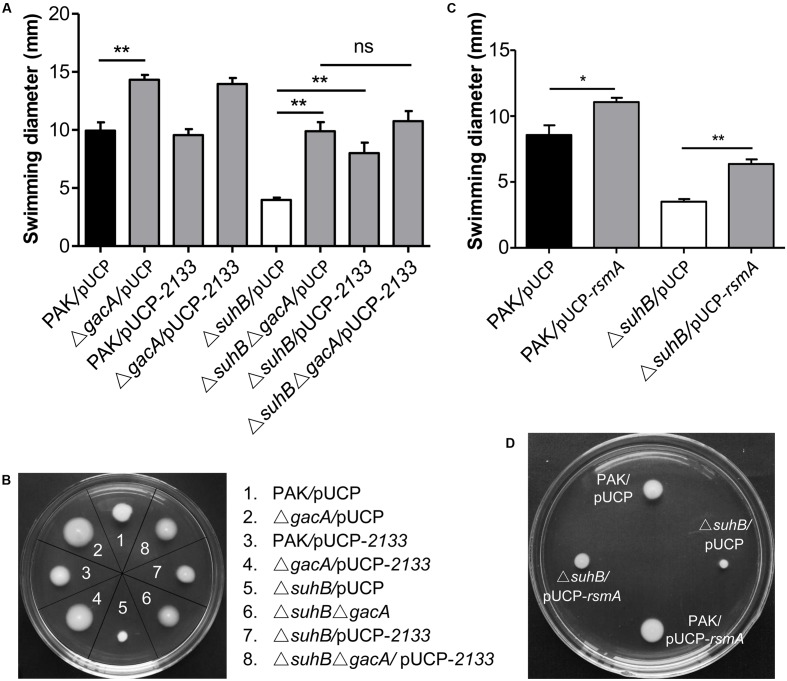
SuhB regulates swimming motility via the Gac/Rsm pathway and c-di-GMP signaling. **(A)** The swimming motility of the indicated strains was analyzed on tryptone soft agar plates (0.3% [w/v] agar). The plasmid pUCP-*2133* allowed overexpression of the phosphodiesterase PA2133, which has an activity for degrading c-di-GMP in *Pseudomonas aeruginosa*. The data represent the average ± SD from at least three different experiments each carried out in triplicate. Significance was determined with a one-way ANOVA followed by Tukey’s multiple comparison. (ns, not significant; ^∗∗^*P* < 0.01). **(B)** Swimming phenotype of the indicated strains. The name of strains used is indicated on the right. Shown is a representative swim plate for each strain. **(C)** Influence of *rsmA* overexpression in PAK wild-type and Δ*suhB* strains on swimming motility. The pUCP-*rsmA* allowed overexpression of the *rsmA* gene cloned into the pUCP20 vector. Each experiment was repeated three times. The error bars indicate standard deviations. (^∗^*P* < 0.05, ^∗∗^*P* < 0.01). **(D)** Swimming motility of PAK wild-type strain and Δ*suhB* harboring the empty vector pUCP20 or pUCP-*rsmA*. Shown are representative from at least three repetitions.

We have previously shown that *suhB* mutation leads to the upregulation of GacA and its downstream small RNAs, RsmY, and RsmZ (Li et al., 2013). Furthermore, we have shown that SuhB regulates the expression of RsmY/RsmZ through GacA (Li et al., 2013). Since the Gac system negatively regulates motility in pseudomonads (Heurlier et al., 2004; Navazo et al., 2009), we hypothesized that the Gac system could be implicated in the repression of swimming motility in the *suhB* mutant. Consequently, the effect of *gacA* mutation on swimming motility was tested. As shown in **Figures 2A,B**, inactivation of *gacA* in wild-type strain PAK resulted in small, but significant increases in swimming motility, while mutation of *gacA* in the *suhB* mutant restored swimming motility to nearly wild-levels. Similar results were obtained when both *rsmY* and *rsmZ* were simultaneous deleted in the *suhB* mutant (Supplementary Figures S1A,B). These results suggest that GacA and RsmY/Z are involved in the SuhB-mediated regulation of swimming motility.

Since the major role of RsmY and RsmZ is to antagonize the RNA binding regulator RsmA, we therefore asked whether the observed motility defect of *suhB* mutant is linked to reduced RsmA levels and can be rescued by elevating levels of this protein. As expected, while plasmid overexpression of RsmA under the control of the *lac* promoter (pUCP-*rsmA*) in wild-type PAK resulted in enhanced motility, multicopy expression of *rsmA* partly rescued the swimming motility of the Δ*suhB* mutant (**Figures 2C,D**), indicating that RsmA is responsible for the *suhB* mutant phenotype. Together, these findings indicated that the regulation of motility by SuhB acted, at least partially, through the GacA-RsmY/Z-RsmA regulatory pathway.

### The *suhB* Mutant Displays Elevated Levels of c-di-GMP

**FIGURE 3:**
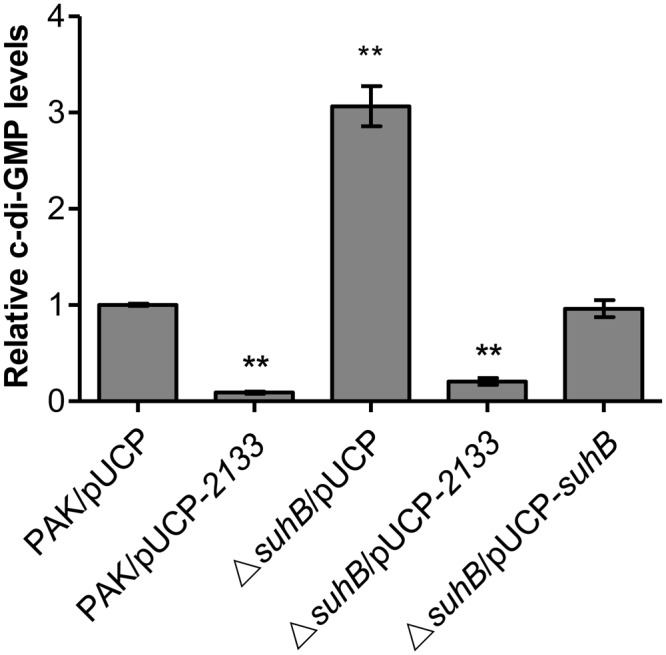
Relative intracellular levels of c-di-GMP measured with the transcriptional P*cdrA-lacZ* reporter. The levels of c-di-GMP in PAK carrying the vector control were set to 1. At least three independent experiments were performed. Error bars represent the standard deviations, and statistically significant changes are indicated (^∗∗^*P* < 0.01, Student’s *t*-test).

In *P. aeruginosa*, mutation in either *suhB* or *retS* results in similar phenotypes from motility to biofilm formation, and SuhB-mediated regulatory pathways have been demonstrated to partially overlap those under the control of RetS (Goodman et al., 2004; Coggan and Wolfgang, 2012; Li et al., 2013). The *retS* mutant displays elevated levels of c-di-GMP, and RetS has been demonstrated to switch the T3SS/T6SS via c-di-GMP signaling (Moscoso et al., 2011). Based on these studies, we hypothesized that loss of *suhB* might also result in an increase in intracellular c-di-GMP levels. To address this, we compared the levels of c-di-GMP between wild-type PAK and the *suhB* mutant by using a transcriptional P*cdrA-lacZ* reporter system that responds to c-di-GMP (Rybtke et al., 2012). As shown in **Figure 3**, the *suhB* mutant displays a ∼3-fold increase in c-di-GMP levels as compared with the wild-type strain. When the *suhB* mutant was complemented with the *suhB* gene, the intracellular c-di-GMP was restored to wild-type levels, indicating that SuhB modulates intracellular c-di-GMP concentrations.

### The Motility-Deficient and Hyperbiofilm Phenotype of the *suhB* Mutant Is c-di-GMP-Dependent

Since c-di-GMP signaling has been implicated in regulating the transition between motile and sessile lifestyles, and mutation of *suhB* results in increased levels of c-di-GMP, we questioned whether the phenotypes of a *suhB* mutant were correlated with a variation in intracellular c-di-GMP levels. If the elevated level of c-di-GMP was responsible for the *suhB* mutant phenotypes, then reducing intracellular c-di-GMP should reverse these phenotypes. To evaluate this possibility, a plasmid expressing a phosphodiesterase (PDE) PA2133 (pUCP-*2133*) or the empty vector pUCP20 was introduced into the *suhB* mutant. As shown in **Figures 2A,B**, overexpression of PA2133 in the *suhB* mutant partially restored swimming motility to wild-type levels. Moreover, we found that both PAK and Δ*suhB* carrying the PA2133 expression vector exhibited very low to undetectable levels of c-di-GMP (**Figure 3**), suggesting that the motility defect of the Δ*suhB* mutant can be partly attributed to the increases in intracellular c-di-GMP concentrations.

Given that both Gac-Rsm pathway and c-di-GMP were responsible for the motility inhibition in *suhB* mutant, we asked whether the two signaling networks act in parallel or there was a link between them. Whereas deletion of *gacA* or overexpressing PA2133 in Δ*suhB* resulted in significant increases in swimming motility, the effects were not additive when PA2133 was overexpressed in the *suhB gacA* double mutant cells (**Figures 2A,B**). Δ*suhB*Δ*gacA* mutant overexpressing PA2133 had swimming phenotypes that were comparable to those carrying the vector control (**Figures 2A,B**). The same phenomenon was observed in the Δ*suhB*Δ*rsmY*Δ*rsmZ* mutant strains (Supplementary Figures S1A,B). These findings indicated that the presence of GacA and sRNAs might be required for the c-di-GMP-mediated regulation of swimming motility in the *suhB* mutant.

**FIGURE 4:**
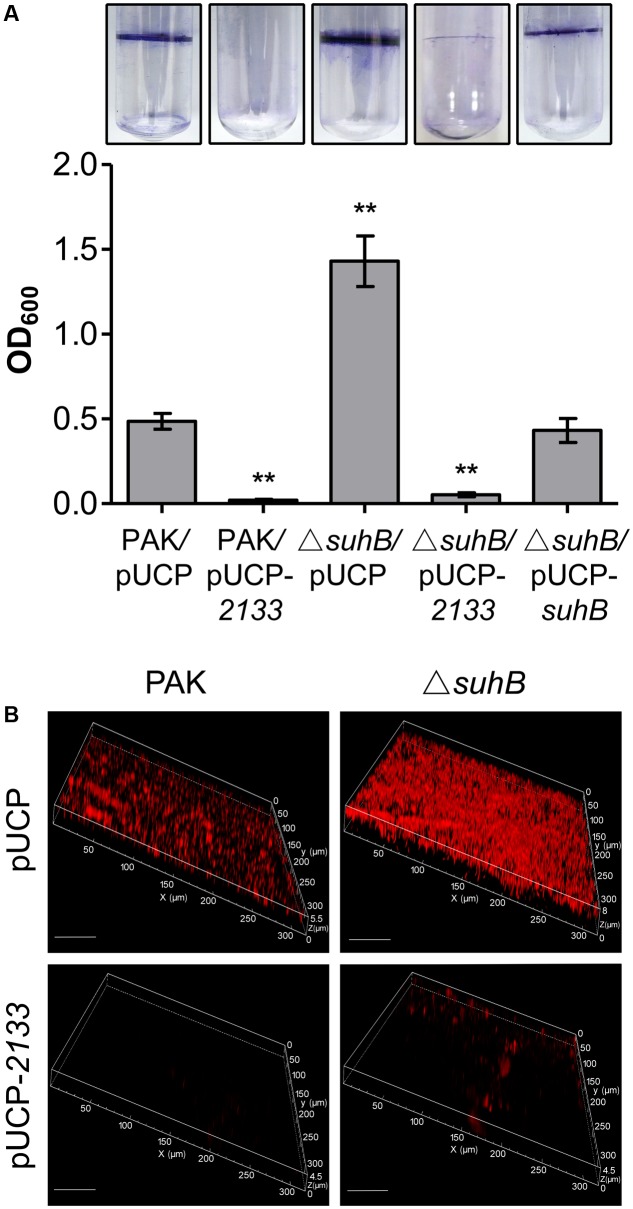
Degradation of c-di-GMP reverses the hyperbiofilm phenotype of a *suhB* mutant. **(A)** Glass tube assay showing biofilm formation (upper part). Quantification of the crystal violet-stained adherence ring formed in the glass tube (lower part). Each experiment was repeated three times. The error bars indicate standard deviations. ^∗∗^*P* < 0.01 compared to WT PAK by Student’s *t*-test. The name of the tested strain is indicated under each bar. The pUCP-*2133* allowed expression of the phosphodiesterase (PDE) PA2133 from the pUCP20 vector. **(B)** Confocal laser scanning microscopy (CLSM) images of biofilms of PAK wild-type and Δ*suhB* harboring a control vector or a plasmid (pUCP-*2133*) expressing PA2133. Biofilms were stained with propidium iodide after 48 h growth. White size bars = 50 μm.

Further, we extended our observation to biofilm formation. The biofilm phenotype was tested in glass tubes and quantified using the crystal violet assay as previously described (O’Toole and Kolter, 1998). Consistent with elevated c-di-GMP levels, the Δ*suhB* mutant showed a hyperbiofilm phenotype compared with the wild-type (**Figure 4A**). This enhanced biofilm formation was dramatically abolished upon overexpression of PA2133 (**Figure 4A**), indicating the hyperbiofilm phenotype of the *suhB* mutant is dependent on elevated c-di-GMP. Additionally, CLSM analysis demonstrated that the *suhB* mutant formed thicker biofilms with more biomass than the wild-type strain PAK, while overexpressing PA2133 significantly reduced biofilm biomass and thickness in the *suhB* mutant (**Figure 4B**). Taken together, these results demonstrate that c-di-GMP dysregulation plays an essential role in the impaired swimming motility and enhanced biofilm formation in *suhB* mutants.

### SuhB Strongly Influences the c-di-GMP Signaling Network

Cyclic di-GMP is produced by diguanylate cyclases (DGC) and degraded by phosphodiesterases (PDE). We hypothesized that the increased levels of c-di-GMP in *suhB* mutant could be a consequence of either activation of gene(s) encoding a DGC or repression of gene(s) encoding a PDE. To define the mechanistic basis for c-di-GMP accumulation and to identify potential target c-di-GMP-metabolizing enzymes in the *suhB* mutant, we compared their transcriptional profiles between wild-type PAK and the Δ*suhB* mutant by real-time PCR. Of the 41 genes encoding GGDEF/EAL/HD-GYP domain proteins, we identified 12 genes exhibiting moderate differences in expression levels (**Table 1**). The DGC genes PA4843 (*gcbA*), PA1107 (*roeA*) showed higher levels, and the PDE genes PA5017 (*dipA*), PA4367 (*bifA*) showed lower levels of transcripts in the *suhB* mutant compared to wild-type PAK. It is worth noting that the transcript level of the PDE gene PA2133 was not changed in the Δ*suhB* strain.

**Table 1:** Expression levels of phosphodiesterases (PDE)/diguanylate cyclases (DGC)-related genes in the *suhB* mutant compared to wild-type PAK.

PA number	Gene name	Fold change (*suhB*/wt)	Gene product or function
PA4843	*gcbA*	2.05	GcbA, DGC
PA1107	*roeA*	1.66	RoeA, DGC
PA3702	*wspR*	-1.60	WspR, DGC
PA1120	*tpbB*	-1.68	TpbB, DGC
PA0847	–	-1.81	DGC
PA5487	–	-1.89	DGC
PA0169	*siaD*	-1.95	SiaD, Predicted DGC
PA2818	*arr*	1.81	Aminoglycoside response regulator, predicted PDE
PA2200	–	1.65	PDE
PA2133	–	-1.03	PDE
PA5017	*dipA*	-1.83	DipA, PDE
PA4367	*bifA*	-2.54	BifA, PDE


### GcbA Is Involved in the SuhB-Mediated Regulation of c-di-GMP Levels Contributing to the Transition between Planktonic and Surface-Associated Growth

**FIGURE 5:**
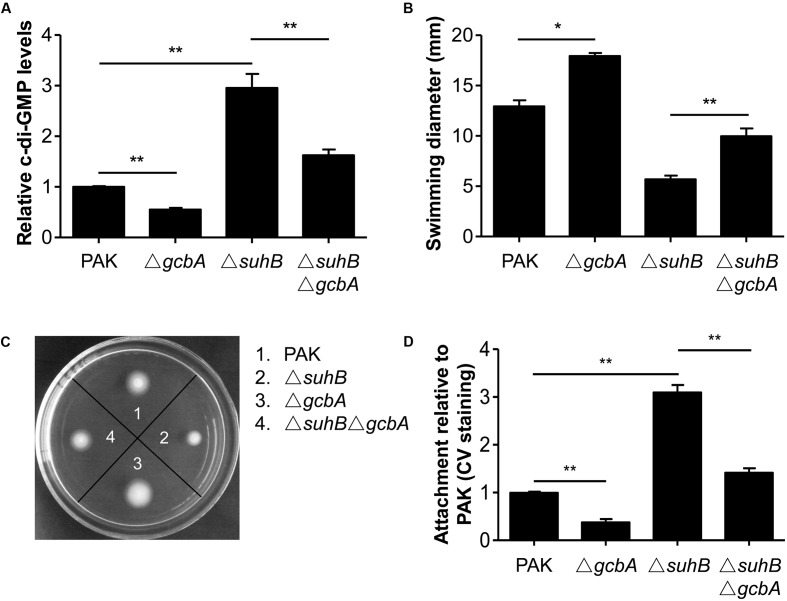
Loss of GcbA reverses the phenotypes of a *suhB* mutant. **(A)** Relative c-di-GMP levels of wild-type PAK, Δ*suhB* and *gcbA* deletion mutants measured with the transcriptional P*cdrA-lacZ* reporter. The levels of c-di-GMP in PAK were set to 1. At least three independent experiments were performed. Error bars represent the standard deviations, and statistically significant changes are indicated (^∗∗^*P* < 0.01, Student’s *t*-test). **(B,C)** Results of swimming motility assay performed on 0.3% tryptone agar plates. **(D)** Evaluation of attachment to a polystyrene surface as determined by crystal violet (CV) staining following 6 h of growth. Attachment of the wild-type PAK has been set to 1. All assays were repeated at least in triplicate. Significance was determined by Student’s *t*-test and is indicated (^∗^*P* < 0.05, ^∗∗^*P* < 0.01).

We next investigated the c-di-GMP-metabolizing genes responsible for the SuhB-mediated regulation of c-di-GMP concentration and hence the motility and biofilm phenotype. In light of the *PA4843* (*gcbA*, also identified as *adcA*) upregulation, and the fact that GcbA was recently characterized to facilitate the transition from planktonic to surface-associated growth by acting as a functional DGC (Jones et al., 2014; Petrova et al., 2014), we engineered a *suhB gcbA* mutant and analyzed the level of c-di-GMP in this strain. As a control, a *gcbA* single mutant was also included. As shown in **Figure 5A**, while inactivation of *gcbA* resulted in a ∼2-fold decrease in c-di-GMP levels of the wild-type, deletion of *gcbA* in the *suhB* background significantly reduced the c-di-GMP concentration of the *suhB* mutant (**Figure 5A**), suggesting that the GcbA diguanylate cyclase is active in the *suhB* mutant and is responsible for the elevated levels of c-di-GMP. Moreover, the Δ*suhB* Δ*gcbA* strain demonstrated significantly elevated swimming motility relative to the *suhB* mutant (**Figures 5B,C**), corresponding to its intermediate level of intracellular c-di-GMP (**Figure 5A**). Finally, inactivation of *gcbA* in Δ*suhB* resulted in a significant reduction in attachment to surfaces relative to the parental *suhB* mutant strain (**Figure 5D**). Altogether, these data suggested GcbA is involved in the elevated levels of c-di-GMP in Δ*suhB* that leads to the transition from planktonic to surface-associated growth.

## Discussion

In this study, we employed different assays to analyze the swimming ability of wild-type and *suhB* mutant strains, including soft agar assays, light microscopy to examine swimming behavior and flagellar reversals, and electron microscopy to observe flagellar structures. Our results extend the observations for the motility of *suhB* mutants and indicate that the absence of *suhB* led to a defect in swimming motility in all growth phases. Since the *suhB* mutant cells exhibited less frequent reorientations in movement, and the transcript of the flagellin gene *fliC* was previously reported to be downregulated in the *suhB* mutant during exponential phase (Li et al., 2013), we suspect that this defect in movement maybe due to decreased expression of flagellar genes or/and a failure in flagellar function of the *suhB* mutant. Nevertheless, TEM ultrastructural studies showed that both the morphology of the flagella and the size of the *suhB* mutant cells were similar to those of WT at the stationary phase. It is worth noting that the rate of flagellar reversals for the *suhB* mutant was significantly reduced compared to WT cells, which suggested a defect in flagellar function of a *suhB* mutant strain.

**FIGURE 6:**
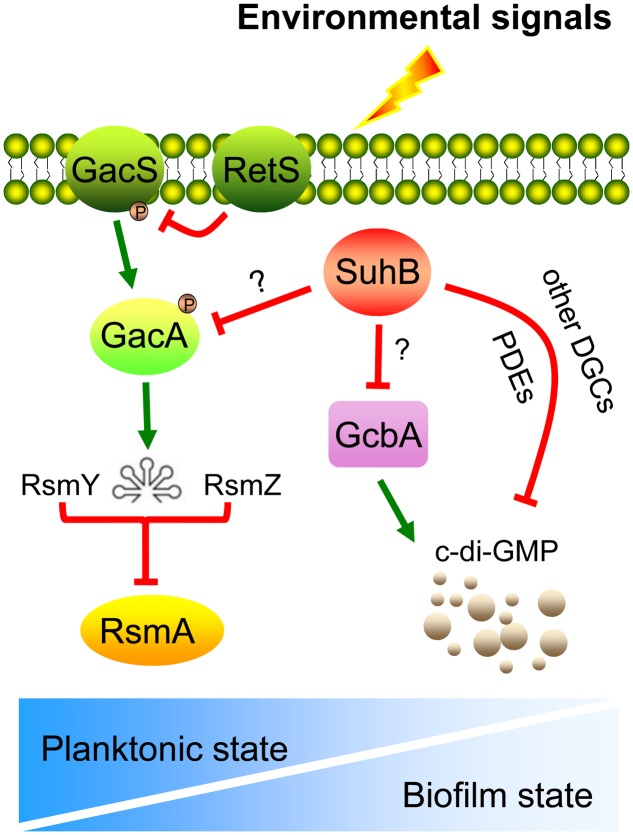
Model for the SuhB-mediated inverse regulation of swimming motility and biofilm formation in *P. aeruginosa*. Upon receipt of the proper environmental signals (such as encounter an appropriate surface or changes in medium viscosity), the free-living bacteria suppress expression of *suhB* and thus levels of GacA and sRNAs, RsmY and RsmZ are elevated, which in turn antagonize the regulatory activity of RsmA, resulting in a shift toward a communal biofilm-associated phenotype. In addition, the intracellular c-di-GMP levels are also increased through activation of the DGC GcbA. We hypothesize that this c-di-GMP pool may also be impacted by the action of additional DGCs or PDEs, acting as a signal to the downstream constituents of the SuhB pathway. The relationship between RetS and GacS-GacA pathway as well as titration of RsmA by RsmY and RsmZ are demonstrated in previously published data (Goodman et al., 2009). The gradient of the blue color in the bottom panel represents the switch between planktonic and biofilm lifestyles.

Defects in swimming motility because of abnormal synthesis and function of flagella could arise from defects in the signal TCS (Rashid and Kornberg, 2000). By engineering mutants with deletions of *gacA* and *rsmY rsmZ*, we were able to conclude that the activity of the Gac/Rsm cascade is directly responsible for the motility inhibition observed in *suhB* mutant. This scenario was further corroborated by overexpression of RsmA, which mimics the phenotypes of the *gacA* and *rsmY rsmZ* mutants. These results, along with our previous observation that GacA and RsmY/Z contribute to the hyperbiofilm phenotype in the *suhB* mutant (Li et al., 2013), support a model wherein the GacA-RsmY/Z-RsmA pathway may participate in SuhB-mediated opposite regulation of swimming motility and biofilm formation (**Figure 6**).

In *P. fluorescens* F113, a link between the Gac system and flagella biosynthesis has recently been described in which it exerts a negative regulation on swimming motility through downregulation of the *fleQ* gene and FliC flagellin production during exponential phase but not during stationary phase (Martinez-Granero et al., 2012). Analogous to this, in *P. aeruginosa* PAK, the expression of FleQ and FleQ-regulated flagellum genes (e.g., *fleS*, *fleR*, and *fliC*) were not affected as revealed by a transcriptome study of the regulon of RsmA using cultures in the stationary phase (Brencic and Lory, 2009). Previous work in our laboratory demonstrated that *suhB* mutation led to the upregulation of GacA and the two small RNAs, RsmY, and RsmZ, whereas the *fliC* transcription was downregulated during exponential phase (Li et al., 2013; Shi et al., 2015). Given the highly conservative of the Gac/Rsm cascade (Lapouge et al., 2008), and assuming the same might be true in *P. aeruginosa*, the swimming motility defects of the *suhB* mutant may be explained on the same basis as in *P. fluorescens* F113.

Besides the Gac/Rsm pathway, the contribution of c-di-GMP to inhibiting motility and activating biofilm formation has been widely demonstrated (Hickman et al., 2005; Tamayo et al., 2007; Hengge, 2009; Ha and O’Toole, 2015). The fact that the levels of c-di-GMP are increased in the *suhB* mutant and artificially degradation of c-di-GMP in Δ*suhB* reversed the behaviors of this strain, suggest the phenotypes of the *suhB* mutant correlate with an increase in intracellular levels of c-di-GMP, thus providing an additional level of control. Using Real-time PCR, we demonstrated that SuhB regulates the expression of several c-di-GMP metabolizing genes, and one of these enzymes, GcbA, was identified to be a target of SuhB. GcbA was recently found to promote initial attachment to surfaces via regulation of flagellum-driven motility by suppressing flagellar reversals (Petrova et al., 2014). It is thus possible to suggest that SuhB-dependent control of motility-to-biofilm transition might act through GcbA to reduce reversal rates and thus increase the time of interaction between cells and the surface, which increases the likelihood of irreversible attachment, the committed step toward biofilm formation (Caiazza et al., 2007; Wolfe and Visick, 2008). However, moderate fold changes were also observed in expression levels of other DGC and PDE coding genes between the wild-type and *suhB* mutant. Since the c-di-GMP levels could only be restored partially in the Δ*suhB* Δ*gcbA* strain, the involvement of additional DGCs or PDEs in the SuhB-mediated regulation could not be excluded (**Figure 6**). The presence of more than one gene, with each contributing to a part of the c-di-GMP metabolism, may explain the moderate changes of most c-di-GMP metabolizing genes in the *suhB* mutant.

Recent evidence has established a link between c-di-GMP signaling and the Gac/Rsm cascade (Moscoso et al., 2011; Martinez-Granero et al., 2012; Moscoso et al., 2014). The *P. fluorescens* Gac/Rsm system and the cytoplasmic c-di-GMP receptor SadB have been shown to converge on AlgU and the transcriptional regulator FleQ to ultimately regulate motility and surface attachment (Navazo et al., 2009; Martinez-Granero et al., 2012). In *P. aeruginosa*, direct evidence came from the observation that a *retS* mutant displays elevated levels of c-di-GMP and that the c-di-GMP-induced T3SS/T6SS switch was found to require the sRNAs RsmY and RsmZ (Moscoso et al., 2011). Later on, the detailed mechanisms of the link were elucidated: SadC, a DGC whose production is repressed by RsmA, is a central player in the Gac/Rsm-mediated regulation of biofilm formation (Moscoso et al., 2014). However, *P. aeruginosa* SadB and SadC were found to exclusively regulate biofilm formation and swarming but not swimming (Sauer et al., 2002; Caiazza et al., 2007). Intriguingly, a recent study provides evidence of a link between GcbA and RsmZ in the regulation of the motile-sessile switch (Petrova et al., 2014). In this case, GcbA levels were found to positively correlate with RsmZ abundance, and the regulation of attachment and motility by GcbA is dependent on RsmZ, with c-di-GMP synthesized by GcbA potentially contributing to the regulation of RsmZ levels (Petrova et al., 2014). Our results indicated a contribution of Gac/Rsm signaling to the c-di-GMP-mediated regulation of swimming motility in Δ*suhB*. However, the exact connection between these systems remains unclear. One possibility is that GcbA partly contributes to a pool of available c-di-GMP in Δ*suhB* to regulate motility through RsmZ, which is consistent with the above mechanism and the observation that loss of *gcbA* in Δ*suhB* partially rescued the *suhB* mutant phenotypes. Yet, the involvement of other components could not be eliminated. The molecular details of SuhB-mediated c-di-GMP turnover and the corresponding downstream targets, the mechanisms by which SuhB exerts its effects on the Gac/Rsm cascade, as well as the hierarchical relationship between these regulatory systems will be the subject of future investigations.

A model summarizing the findings from this study is shown in **Figure 6**. In wild-type PAK, the expression level of *suhB* was significantly reduced under sessile growth compared to that under planktonic growth (Supplementary Figure S2). We propose that the SuhB protein coordinates the activities of Gac/Rsm cascade and c-di-GMP, to reciprocally influence swimming motility and biofilm formation as *P. aeruginosa* transitions from a planktonic to a surface-associated lifestyle. In addition, it appears that the two major regulatory mechanisms are interconnected to each other, which reflect the importance of appropriately modulating cellular behaviors, and could allow for a quickly and energy-efficient adaptation in response to changing conditions.

## Author Contributions

Conceived and designed the experiments: KL, SJ, WW, and QB. Performed the experiments: KL, GY, AD, PpL, LZ, PzL, and TX. Analyzed the data: KL, GY, AD, and QB. Wrote the paper: KL, GY, and QB.

## Conflict of Interest Statement

The authors declare that the research was conducted in the absence of any commercial or financial relationships that could be construed as a potential conflict of interest.
