# Supplementary material for: SuhB Regulates the Motile-Sessile Switch in Pseudomonas aeruginosa through the Gac/Rsm Pathway and c-di-GMP Signaling
Source: Front Microbiol. 2017 Jun 8;8:1045. doi: 10.3389/fmicb.2017.01045 (PMC5462983; doi:10.3389/fmicb.2017.01045)
Supplement: Supplementary file 1 [file Data_Sheet_1.PDF]

<u>***Supplementary Material***</u>

SuhB Regulates the Motile-Sessile Switch in *Pseudomonas aeruginosa* through the Gac/Rsm Pathway and c-di-GMP Signalling

Kewei Li [1] , Guangjian Yang [1] , Alexander B. Debru [1] , Pingping Li [1] , Li Zong [1] , Peizhen Li [1] , Teng Xu [1] , Weihui Wu [2 *], Shouguang Jin [2, 3 *] and Qiyu Bao [1 *]

*** Correspondence:** *Qiyu Bao: baoqy@genomics.cn; Shouguang Jin: sjin@ufl.edu; Weihui Wu: wuweihui@nankai.edu.cn*

1 Supplementary Figures and Tables

1.1 Supplementary Figures

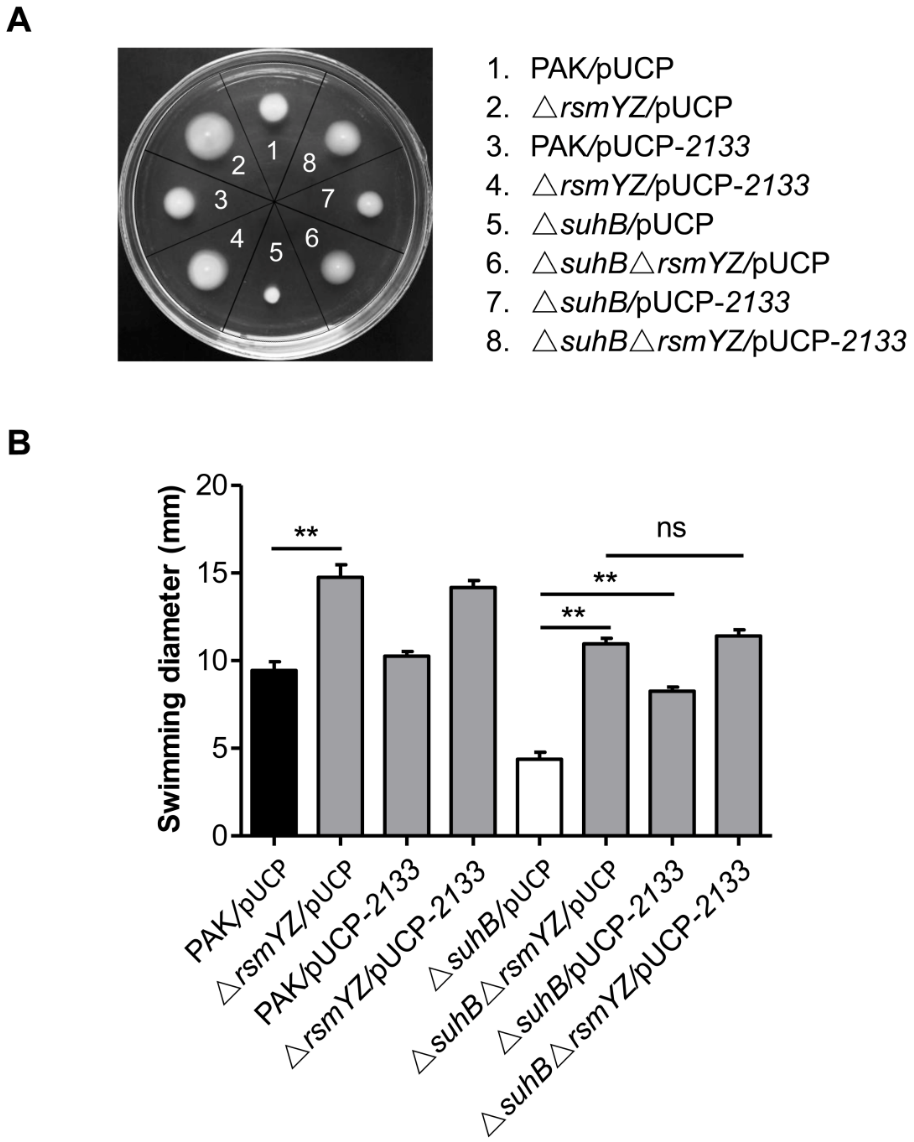

Figure S1. RsmY/Z and c-di-GMP signaling are involved in the SuhB-mediated regulation of swimming motility. (A) The swimming motility of indicated strains was analyzed on 0.3% (w/v) tryptone soft agar plates. The plasmid pUCP-*2133* allowed overexpression of the phosphodiesterase PA2133, which has an activity for degrading c-di-GMP in *P. aeruginosa*. The name of strains used is indicated on the right. Shown is a representative swim plate for each strain. **(B)** Diameters of the swimming zones of indicated strains. Data show the average ± SD from at least three different experiments each carried out in triplicate. Significance was determined with a one-way ANOVA followed by Turkey's multiple comparison. (ns, not significant; **, $P < 0.01$).

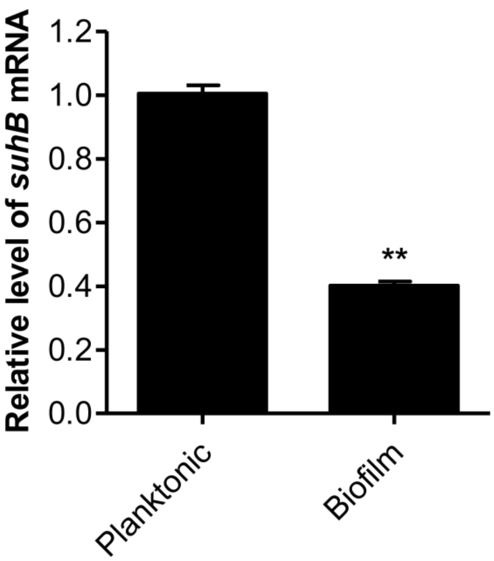

Figure S2. The expression patterns of *suhB* in planktonic and biofilm cells. WT PAK was inoculated into wells in a 96-well plate and grown at 37°C for 24 h. Planktonic bacteria in LB were taken from each well. RNAs from planktonic and sessile bacterial cells were purified and relative mRNA levels of *suhB* were determined by real-time PCR. mRNA levels of *suhB* are reported relative to the abundance in planktonically grown PAK cells. **, significantly different from expression in planktonically grown PAK cells ($P < 0.01$).

1.2 Supplementary Tables

Table S1. Strains and plasmids used in this study.

Strain or plasmid	Relevant genotype or description	Source or reference
P. aeruginosa		
PAK	Wild-type clinical isolate	David Bradley
Δ*suhB*	PAK with a *suhB* deletion	(Li et al., 2013)
Δ*gacA*	PAK with a *gacA* deletion	(Li et al., 2013)
Δ*rsmYZ*	PAK with deletions of *rsmY* and *rsmZ*	(Li et al., 2013)
Δ*suhB*Δ*gacA*	PAK Δ*suhB* with a *gacA* deletion	(Li et al., 2013)
Δ*suhB*Δ*rsmYZ*	PAK Δ*suhB* with deletions of *rsmY* and *rsmZ*	(Li et al., 2013)
Δ*gcbA*	PAK with a *gcbA* deletion	This study
Δ*suhB*Δ*gcbA*	PAK Δ*suhB* with a *gcbA* deletion	This study
Escherichia coli		
DH5α	φ80d*lacZ*ΔM15 Δ(*lacZYA-argF*)*U169 recA1 endA1 hsdR17*(r_K^- m_K^-) *supE44 thi-1 gyrA relA1*	(Taylor et al., 1993)
S17-1	*thi pro hdsR hdsM*$^+$ *recA*; chromosomal insertion of RP4-2 (Tc::Mu Km::Tn7)	Jin lab
Plasmids		
pEX18Tc	Gene replacement vector; Tc^r, *oriT*$^+$, *sacB*$^+$	Jin lab
pUCP20	Shuttle vector between *E. coli* and *P. aeruginosa;* Ap^r	Jin lab
pUCP-*suhB*	*suhB* cloned into pUCP20 driven by *lac* promoter; Ap^r	(Li et al., 2013)
pUCP-*zsGreen1*	*zsGreen1* cloned into pUCP20 driven by *lac* promoter; Ap^r	This study
pUCP-*rsmA*	*rsmA* cloned into pUCP20 driven by *lac* promoter; Ap^r	This study
P*cdrA-lacZ*	*cdrA* promoter of PAK fused to promoterless *lacZ* on pDN19*lacZ*Ω; Sp^r, Sm^r, Tc^r	This study
pUCP-*2133*	*PA2133* cloned into pUCP20 driven by *lac* promoter; Ap^r	This study
pEX-*gcbA*	*gcbA* deletion plasmid, pEX18Tc with 822 bps upstream and 1038 bps downstream of *gcbA*; Tc^r	This study

Table S2. Primers used in this study.

Primer	Sequence (5' to 3')	Use
pEX-*gcbA*-up-F	TGGACTGGTACCGCGAACGGGCGAAATCCTTG	Constructing *gcbA* mutant
pEX-*gcbA*-up-R	GGTCCAGGATCCGGTCATCGTGCTCGGTCATC	
pEX-*gcbA*-down-F	TCGAGGATCCTCGTCCCGACGCTCGATAATGG	
pEX-*gcbA*-down-R	ATCCCAAGCTTGATCAGGCGTACCTGGCCTTG	
pUCP-*zsGreen1*-F	CGCGGATCCATTTCACACAGGAGATATCATATGGCCCAGTCCAAGCACGGC	Constructing pUCP-*zsGreen1* plasmid
pUCP-*zsGreen1*-R	CCCAAGCTTTCAGGGCAAGGCGGAGCCGG	
pUCP-*rsmA*-F	AGACTGGAATTCTGAAATATTCGCGTGAGGAG	Constructing pUCP-*rsmA* plasmid
pUCP-*rsmA*-R	GGCGCGGATCCTACCCATCTTTACCCCGTTTG	
cdrA-LacZ-F	GGCGTGGAATTCCCATGGCAGTTGGCGACGAC	Constructing P*cdrA*-*lacZ* plasmid
cdrA-LacZ-R	CGGACGGGATCCGAAAATCTCCCTATCTGCGT	
pUCP-*2133*-F	GGCGTCGGATCCTCACACAGGAAACTACAGTGAACGGTTCCCCAC	Constructing pUCP-*2133* plasmid
pUCP-*2133*-R	GCTCCCAAGCTTGGAAGGCTGATTGCTCTGTT	
gcbA -F	CACCGAGATGGTCCTCAACA	Real time PCR
gcbA -R	GCGGACAGGTAGATGATCGG	
roeA -F	GCCAGGAAATCATGTACGCC	Real time PCR
roeA -R	GCCGAAGCCTATGATCCCTG	
wspR -F	TCTCCAACCGTCGTCACTTC	Real time PCR
wspR -R	GGCCGAAGGTGTCGTTGTA	
tpbB -F	TCCAGCGAAGAGGTCTCCAG	Real time PCR
tpbB -R	CCTCCACCGAACCGATCTTC	

Table S2., continued

Primer	Sequence (5' to 3')	Use
PA0847-F	TCTGGCTATTCCTGCCGTTG	Real time PCR
PA0847-R	ATGTCCAGATGGCGCTTCAA	
PA5487-F	GATTTCATCGCCCGTTTCGG	Real time PCR
PA5487-R	GCTCGCCCTTGAAATGGAAC	
siaD-F	CTCAGGGAGGAGAACGAACG	Real time PCR
siaD-R	AGGTCGTATTCGCGTAGCTC	
arr-F	TATCTGGCGCACCTGTTCTG	Real time PCR
arr-R	CAGGGGGATGAAGATGTCCG	
PA2200-F	ATCCTGCCGATACTGCTCAC	Real time PCR
PA2200-R	AGCATTTCCAGATCCCGGTC	
PA2133-F	CCGGGAGTTCATCCGACG	Real time PCR
PA2133-R	GACGGGCATCGAGAACGAAG	
dipA-F	TCAAGCTCAAGGTAGTCGCC	Real time PCR
dipA-R	CTGGTATTGAGCAGGTCGCT	
bifA-F	CGAGCAGTACACCTACCAGC	Real time PCR
bifA-R	TCGTAGGGTTGCTCGATGTC	
suhB-F	AACTGATTTTCCGCTCCAT	Real time PCR
suhB-R	TTCGGTGACGTAGTCCTT	
